# A comparative quantitative & qualitative assessment in orthodontic treatment of white spot lesion treated with 3 different commercially available materials - *In vitro* study

**DOI:** 10.4317/jced.56044

**Published:** 2019-09-01

**Authors:** Pooja Yadav, Hina Desai, Kalpesh Patel, Nikunj Patel, Shreya Iyengar

**Affiliations:** 1 3rd Year PG, Department Of Orthodontics & Dentofacial Orthopaedics, Manubhai Patel Dental College & Oral Research Institiute, Vadodara; 2 Vice Dean & Head Of Department, Department Of Orthodontics & Dentofacial Orthopaedics, Manubhai Patel Dental College, Vadodara; 3 Professor, Department Of Orthodontics & Dentofacial Orthopaedics, Manubhai Patel Dental College & Oral Research Institiute, Vadodara; 4 Senior Lecturer, Department Of Orthodontics & Dentofacial Orthopaedics, Manubhai Patel Dental College & Oral Research Institiute, Vadodara; 5 Senior Lecturer, Department Of Orthodontics & Dentofacial Orthopaedics, Manubhai Patel Dental College & Oral Research Institiute, Vadodara

## Abstract

**Background:**

To comparatively evaluate the esthetic improvement of white-spot lesions (WSLs) treated by: BiominF, CPP-ACP paste with fluoride & ICON resin infiltration, using Spectrophotometer & Diagnodent.

**Material and Methods:**

The study was done using 72 sound permanent extracted premolars, divided into four groups (18 teeth per group). After taking the ethical approval the study was commenced. WSLs were created on human premolars and randomly assigned to four groups: Group A: Artificial Saliva, Group B: CPP-ACP with fluoride, Group C: BiominF, Group D: Resin infiltration (Icon). The color change (∆E) of each specimen was measured with a Spectrophotometer (VITA Easy Shade Compact), and fluorescence loss (∆Q) was measured by a laser fluorescence device (DIAGNOdent, Kavo, Biberach, Germany), at different time points after treatment: baseline (0 weeks), 2 weeks, 4 weeks, and 6 weeks.

**Results:**

The ∆E and ∆Q baseline values for the four groups before the treatments did not differ significantly. Icon treatment improved the WSL color significantly and gave the lowest ∆E (5.12± 3.92) & ∆Q (1.64 ±0.72) compared with other treatments at end of 6 weeks (*P*< .01). In the BiominF and CPP-ACP with fluoride treatment groups, ∆Q & ∆E showed significant recovery compared with the baseline values (*P*< .05).

**Conclusions:**

Within the limitations of the study, it can be concluded that all the three remineralizing agents used in the study could effectively remineralize artificial enamel caries and showed improvement in color change and fluoresence as compared to the baseline. Therefore they can be effectively used for the treatment of the white spot lesions.

** Key words:**White spot lesions(WSL), Resin infiltration (ICON), BiominF, CPP-ACP with fluoride.

## Introduction

A pleasing smile and well aligned teeth are not only important from the cosmetic point of view but also carry positive status in the society. Fixed orthodontic treatment, no doubt, improves the dental aesthetics but white spot lesions (WSL), the silent factor killing the smile, are one of the most common dilemma for orthodontics (1).

White-spot lesions (WSLs) are one of the most prevalent side effects of fixed orthodontic treatments, affecting about 50% of the patients (1). Fejerskov O *et al.* reported that estimates of the prevalence of White Spot Lesion arising during fixed orthodontic treatment range widely from 2% to 96% (2).

Once WSLs occur, treatment usually includes noninvasive measures, such as remineralization or resin infiltration, and invasive measures, such as microabrasion or the placement of veneers or crowns. Invasive measures involve removal of enamel to various degrees. Clinicians therefore tend to prefer to start treatment with a relatively noninvasive approach (3). Recommendation of fluoridated toothpaste at the time of orthodontic treatment is very common by orthodontist. Commonly used fluoridated toothpastes are NaF, SnF2, monofluorophosphate or a combination of these. Newer remineralising agents such as CPP-ACP (casein phosphopeptide amorphous calcium phosphate) and Biomin F have also been recently introduced (4).

CPP-ACP along with fluoride have proved to give better results and now are commercially available (4). Resin infiltration was developed to arrest caries lesion progression with low-viscous light-curing resins (5). Recent studies have found that resin infiltration could also restore the color of WSLs after orthodontic treatment (6-8). However, the effect of resin infiltration on recovering other aspects of WSLs, such as fluorescence loss is still not clear.

Currently, it is very difficult for clinicians to easily identify which approach works best for WSLs. The purpose of this *in vitro* study is to compare the esthetic improvement of WSLs quantitatively & qualitatively treated by three commonly used methods, CPP-ACP with fluoride, BiominF and resin infiltration using spectrophotometer and Kavo Diagnonent measurements.

## Material and Methods

The present *in vitro* study was conducted at Department of Orthodontics & Dentofacial Orthopaedics, Manubhai Patel Dental College & ORI, Vadodara.

SOURCE OF DATA: The samples (72 sound permanent extracted teeth) were stored in an incubator in Oral Pathology Department at Manubhai Patel Dental College, Hospital & ORI, Vadodara.

Inclusion criteria: The permanent maxillary & mandibular sound premolar teeth which were therapeutically extracted for orthodontic treatment purpose were selected.

Exclusion criteria: Teeth with visible and detectable caries, hypoplastic lesions, stains, existing white spot lesions, cracks and erosion have been excluded from the study. Random sampling method have been used to select the sample group as per the inclusion and exclusion criteria.

Sample Size: Minimum 144 [72 teeth * 2 (buccal & lingual surface) ] samples required for present study to get mean differences ( of loss in fluorescence) by 3.7% with standard deviation of 4.5 at 5% risk & 80% power. So 72 teeth were kept in study.

METHODOLOGY:

The study was done using 72 (18 per group) sound permanent extracted teeth. Caries- free premolars extracted for orthodontic reasons were included in this study. The extracted teeth were stored in normal saline immediately after extraction. The teeth were then thoroughly cleaned of its debris and soft tissues avoiding minimal damage to tooth structure. Specimens were randomly divided into 4 groups, each group having 18 teeth (36 surface): ( Table 1).

**Table 1:**
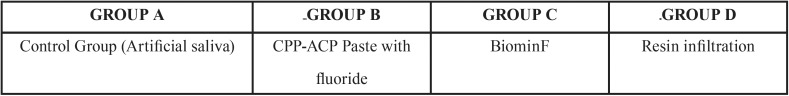
Specimens were randomly divided into 4 groups, each group having 18 teeth (36 surface).

On each extracted tooth (WSLs) were restricted to 3 mm × 3 mm of the enamel surfaces by coating the surrounding enamel surfaces with two layers of fluoride varnish to limit the demineralization to white spot lesion for accurate measurement of the lesion depth (He Yuan *et al.*, Patil *et al.* (9-10).

A demineralizing solution was then prepared. The composition of demineralizing solution is as follows.

**Figure 1:**
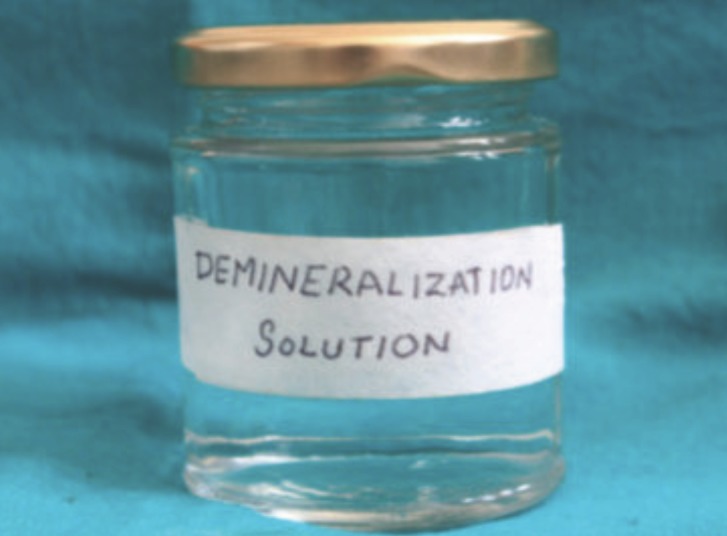
Demineralizing Solution.

Demineralizing solution (Fig. 1)

• 2.2 mM calcium chloride (CaCl2.2H2O) 

• 2.2 mM monosodium phosphate (NaH2PO4.7H2O) 

• 0.05 M lactic acid 

**Figure 2:**
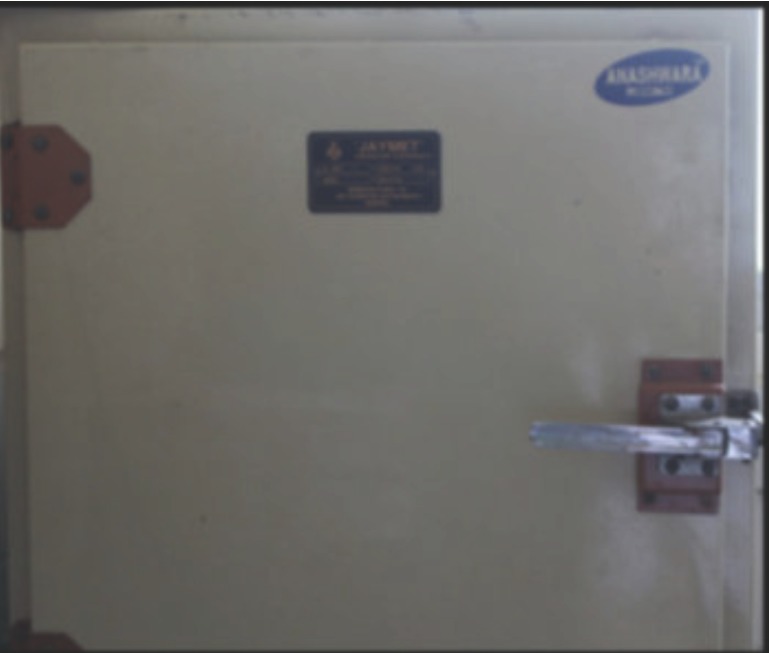
Incubator.

The final pH was adjusted to 4.5 with 50% sodium hydroxide (NaOH). All the samples were then immersed into a glass container containing 150ml of demineralizing solution for 3 weeks at 37 C° using an incubator (Fig. 2).

**Figure 3:**
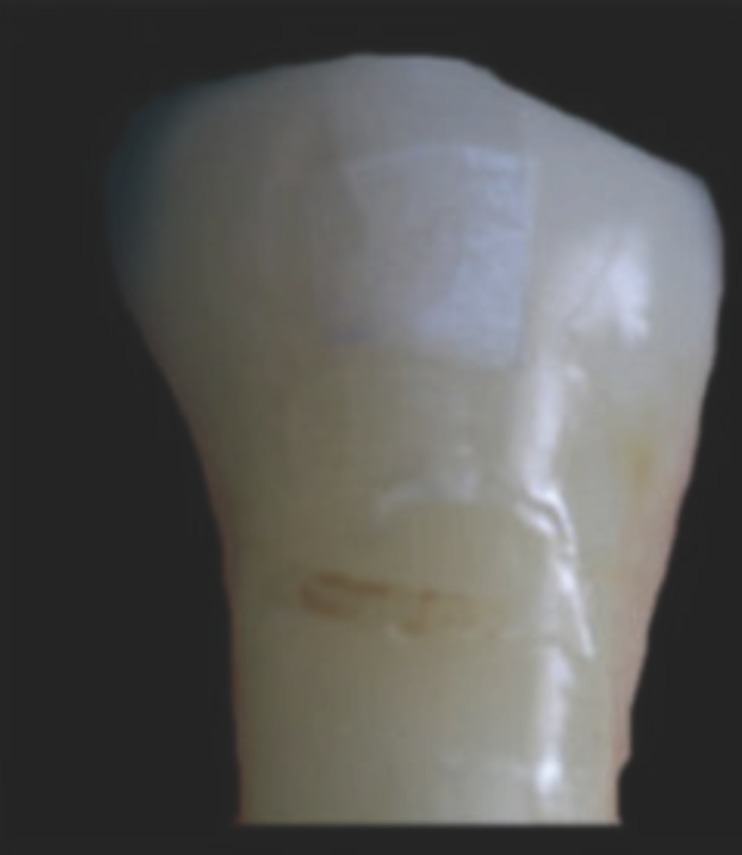
Premolar with white spot lesion.

After this procedure each tooth displayed an artificial WSL of 2 mm × 3 mm (Fig. 3).

After completion of white spot lesion formation, teeth were evaluated with DIAGNOdent® (KaVo, Biberach, Germany) to assess for any surface changes present on the buccal & lingual window. The diagnodent score between 3 - 15 indicated the presence of a subsurface lesion on the tooth surface. Artificial saliva was replenished every 24 h just before immersion of freshly treated samples.

**Figure 4:**
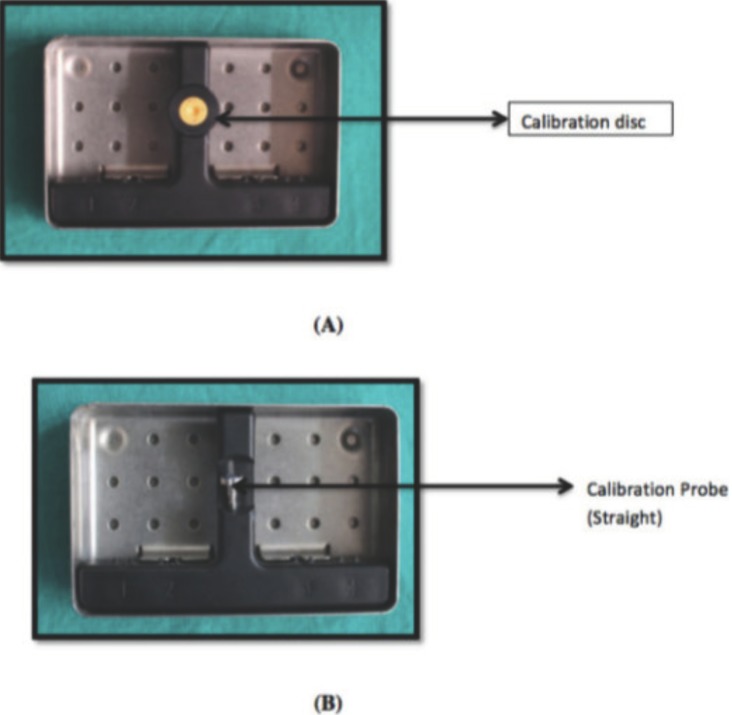
(A) Calibration disc & (B) Calibration probe of diagnodent.

**Figure 5:**
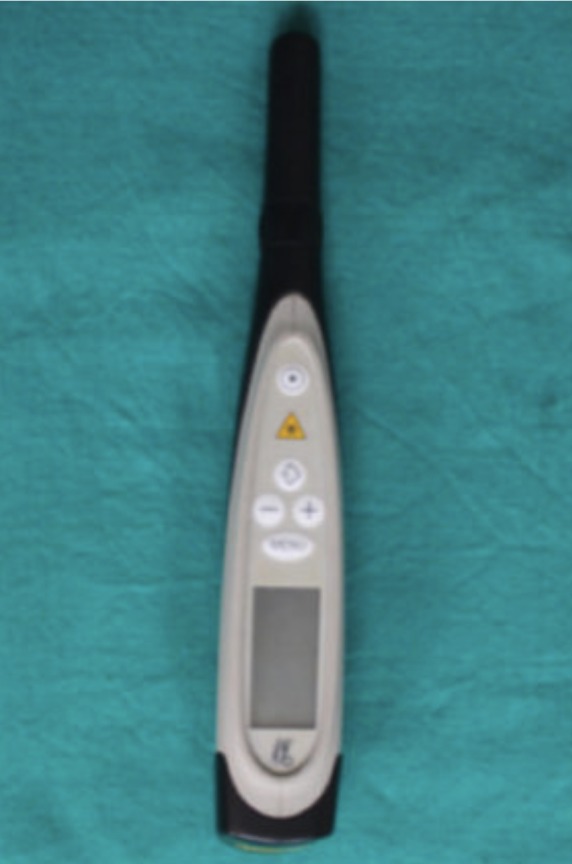
Diagnodent pen (KaVo, Biberach, Germany).

**Figure 6:**
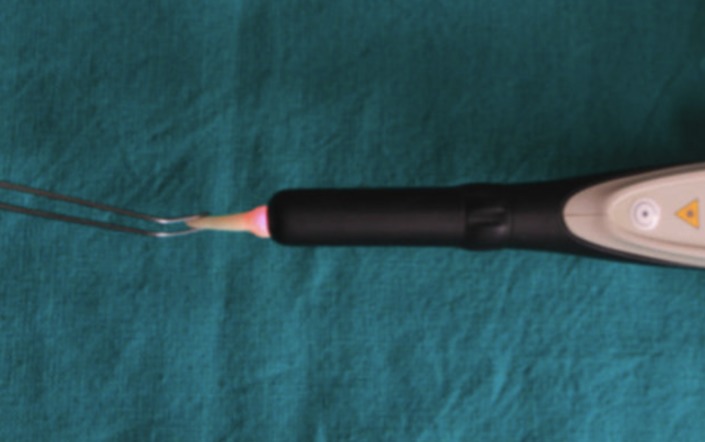
Diagnodent kept perpendicular to the tooth surface.

As recommended by the manufacturer, prior to every measurement session, the instrument was calibrated (Fig. 4). The buccal & lingual window area was carefully scanned by holding the tip in close contact with the tooth surface and tilting the tip around the measuring area in order to collect the fluorescence from all directions. (Figs. 5,6). Samples showing a moment value between 3 and 15 on the digital display were selected. This value showed the presence of a subsurface lesion on the surface of tooth. (Patil, *et al.* (10)).

**Figure 7:**
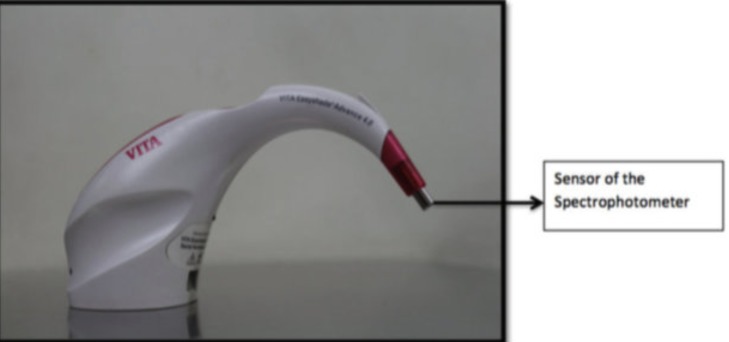
Spectrophotometer (VITA EASYSHADE COMPACT).

The samples were also assessed for color change using spectrophotometer (Fig. 7).

**Figure 8:**
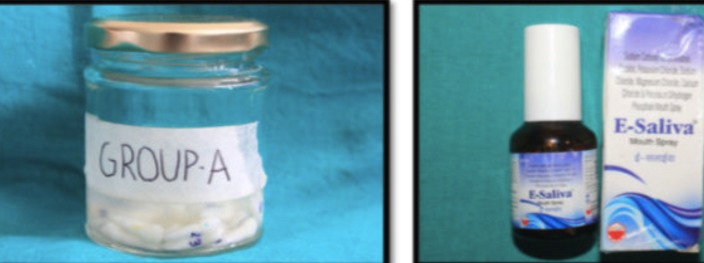
Group A - Artificial Saliva.

In Group A: Teeth were placed in artificial saliva at 37°C (Fig. 8).

**Figure 9:**
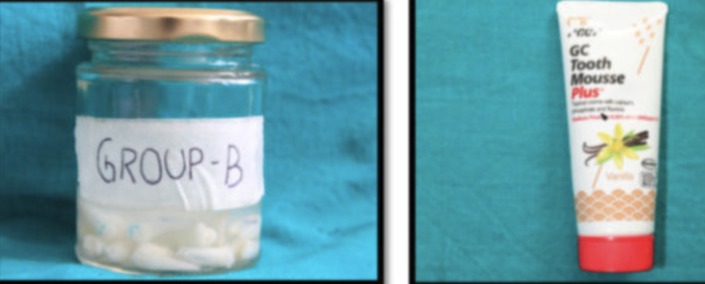
Group B- CPP-ACP Paste with fluoride ( GC Tooth Mousse Plus.

In Group B : on each sample CPP-ACP Paste containing fluoride (GC Tooth Mousse Plus) was applied with a microbrush twice a day for 4 minutes, following which they were placed back in artificial saliva solution without rinsing (Fig. 9).

**Figure 10:**
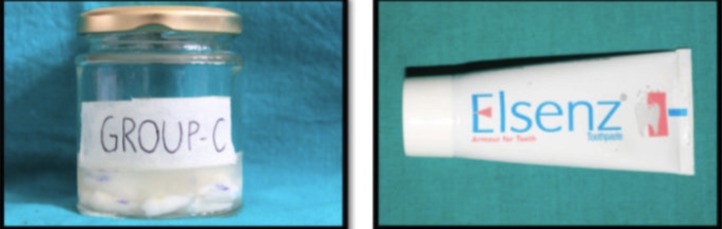
Group C - BiominF (Elsenz) toothpaste.

In Group C: on each sample BiominF (Elsenz) toothpaste was applied with a microbrush twice a day for 4 minutes, following which they were placed back in artificial saliva solution without rinsing (Fig. 10).

**Figure 11:**
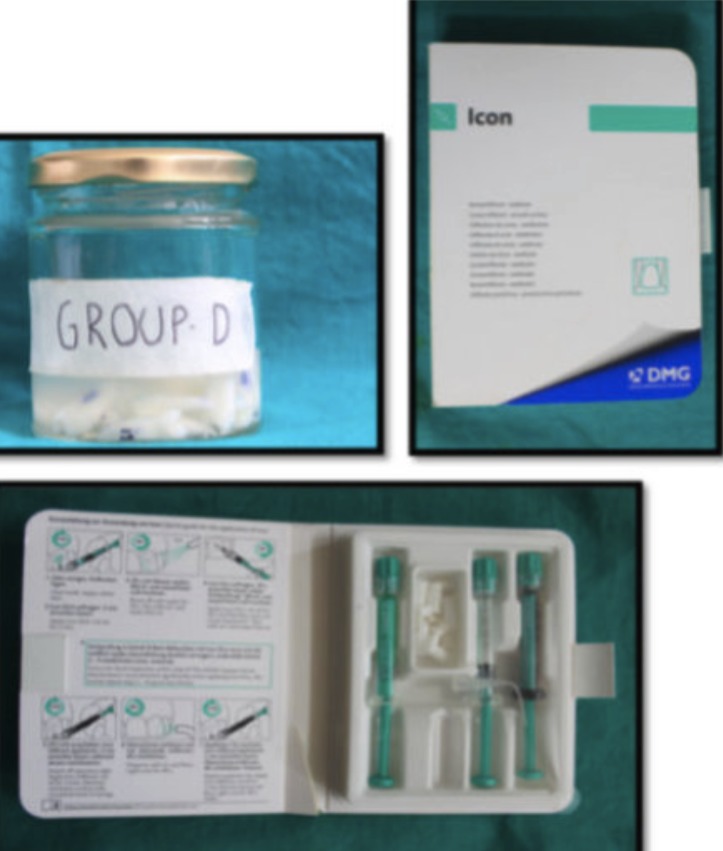
Group D - Resin infiltration (Icon, DMG).

In Group D on each sample the resin infiltration (Icon, DMG) was performed. The White spot lesion was etched for 2 minutes with 15% hydrochloric acid (Icon Etch, DMG) and then rinsed with air-water-spray for 30 seconds. The White spot lesion was desiccated by air-blowing for 10 seconds, followed by application of ethanol (Icon Dry, DMG) for 30 seconds,and air-blowing again for 10 seconds. Then, the resin infiltrant (Icon Infiltrant, DMG) was applied to the WSLs with a sponge applicator provided by the Icon resin infiltration system and was left for 3 minutes. The resin was light-cured for 60 seconds. The infiltration step was repeated once with a penetration time of 60 seconds to infiltrate remaining porosities. The prepared specimens were then polished for 20 seconds according to the instructions, following which they were placed back in artificial saliva solution without rinsing (Fig. 11).

A laser fluorescence device (DIAGNOdent, Kavo, Biberach, Germany) with probe tip B was used. The device was calibrated against a porcelain standard before reading. The probe tip B was kept perpendicular to the tooth surface. The maximum Laser Fluorescence values were recorded for each tooth. After reading each tooth three times by the operator the mean values were obtained.

For measuring the color change using a spectrophotometer ,the readings were recorded three times for each specimen and mean values were obtained using Spectrophotometer (VITA Easy Shade Compact). The values of L* (differences in lightness), a* (green-red coordinate), and b* (blue- yellow coordinate) were recorded for the WSL and the adjacent sound enamel. The loss in fluorescence (∆Q) & color measurements (∆E) were performed at four different treatment time points: baseline (0 weeks), 2 weeks, 4 weeks, and 6 weeks after treatment.

The color measurements & loss in fluorescence were repeated at specified weekly interval to see if, with time after the orthodontic treatment, there was improvement or worsening of the WSLs under conditions mimicking the natural oral environment.

## Results

Observations and results are divided into two parts: Statistical analysis for : (1) DIAGNOdent scores and (2) Color change by Spectrophotometer.

-Statistical Analysis 

The job of data entry, validity checks and formation of desired results (as per analysis plan) was done using statistical package of social sciences (SPSS version 22.0). The level for statistically significanct was set at *P* ≤ 0.05. Statistical analysis was done for intragroup, intergroup between two groups, and multiple group comparison of DIAGNOdent score by using ANOVA test followed by Post Hoc Tuckey test was performed.

**Figure 12:**
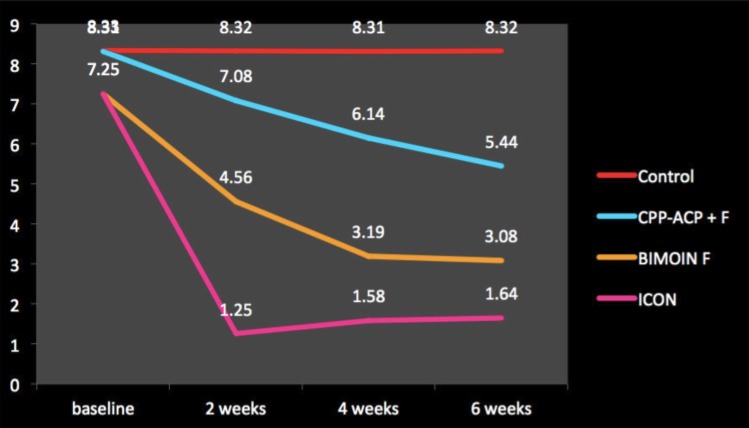
Fluorescence value as measured by Diagnodent at different time periods.

The Figure 12 describes the change in fluorescence from baseline to 6 week after treatment. The mean value of fluorescence at baseline was 8.33 and no improvement was seen from baseline to 6 weeks after treatment. The mean fluorescence value for CCP- ACP paste was 8.31 at baseline and decreased to 7.08 at 2weeks, 6.14 at 4 weeks and 5.44 at 6 weeks. The mean fluorescence value for Biomin F was 7.25 at baseline and decreased to 4.56 at 2weeks, 3.19 at 4 weeks and 3.08 at 6 weeks. The mean fluorescence value for ICON was 7.25 at baseline and decreased to 1.25 at 2weeks, 1.58 at 4 weeks and 1.64 at 6 weeks.

The difference in fluorescence was measured with the help of Diagnodent. The Change in fluorescence from tooth structure decreases from baseline to 6 weeks after treatment. The significant difference was seen among all the treatment groups with maximum improvement after 6 weeks was observed in ICON group followed by Biomn F and CPP- ACP + F paste. Intergroup comparison between groups showed statistically significant improvement in DIAGNOdent scores at each stage in the study.

**Figure 13:**
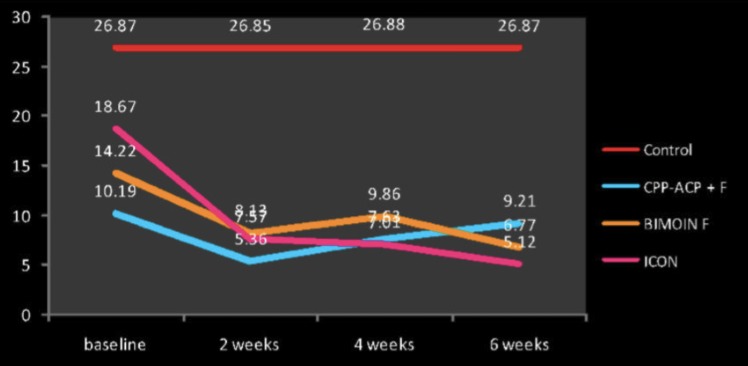
Change in Color (ΔE) as measured by spectrophotometer at different time periods.

The Figure 13 describes the change in color from baseline to 6 week after treatment. The mean values for Color Change at baseline was 26.87 and no improvement was seen from baseline to 6 weeks after treatment. The mean difference in color value for CPP- ACP paste was 10.19 at baseline and decreased to 5.36 at 2weeks, then increased to 7.63 at 4 weeks and finally changes to 6.77 at 6 weeks. The mean difference in color value for Biomin F was 14.22 at baseline and changed to 8.13 at 2weeks, 9.86 at 4 weeks and finally to 6.77 at 6 weeks. The mean Color change for ICON was 18.67 at baseline and decreased to 7.62 at 2weeks, 7.07 at 4 weeks and 5.12 at 6 weeks.

The difference in color (E) was measured with the help of spectrophotometer. The difference in color from tooth structure decreases from baseline to 6 weeks after treatment. The significant decrease was seen among all the treatment groups with minimum difference in color after 6 weeks was observed in ICON group with E at 5.12 followed by Biomn F with difference in color at 6.77 and maximum difference in color from the tooth structure was observed in CPP-ACP paste with 9.21.

## Discussion

This study compared the effects of three commonly used treatment modalities for WSLs against that of a control group (artificial saliva) during a follow-up of 6 weeks after treatment.

At 6 weeks when the diagnodent score was compared to baseline Icon treatment gave the lowest Diagnodent Score (1.64 ±0.72, *P* = 0.01) compared with other treatments. However when compared to 2 weeks & 4 weeks though the diagnodent score at 6 weeks increased it was not significant and showed that the emission of fluorescence similar to that of intact enamel. At 6 weeks Icon treatment showed the lowest value for color change (5.12± 3.92, *P* = 0.01) compared with other treatments. Moreover, the color change at 6 weeks when compared to 2 weeks & 4 weeks reduced the value of ΔE indicating that with time ICON showed even further improvement in color change & was most effective in masking the WSL mimicking the natural enamel color.

Significant decrease of ΔE values were found in the Icon group after the treatment indicating that Icon had the best effectiveness of the three treatments in masking the WSLs and returning the enamel to its natural color. This is because the air or water in the microporosities of WSLs was replaced with resin, leading to less light scattering within the enamel (6). It was found that remineralization of WSLs by the artificial saliva for the BiominF and CPP-ACP + F treatment groups was a slow process because of its dependence on the deposition of calcium ions (11,12). In contrast, the low-viscous resin could penetrate into deeper lesions and improve the esthetic appearance of the WSL immediately after treatment. That explains why the ΔQ immediately showed significant recovery for the Icon group, whereas there was improvement in ΔQ only after 4 weeks of remineralization in the BiominF and CPP- ACP + F groups in this study.

Fluoride is used extensively to promote the remineralization of WSLs (13,14), and a daily NaF mouth rinse might reduce the occurrence and severity of WSLs during orthodontic treatment (15); however, the esthetic improvement of WSLs treated by fluoride is poorly understood. In this study, we found that the color of WSLs cannot be recovered using BiominF treatment. Although the BiominF group showed some improvement of ΔQ 4 weeks after treatment, there was no significant difference in ΔQ with the control group. Therefore, we cannot attribute the ΔQ recovery to the application of BiominF; it is likely due to remineralization by the artificial saliva.

It has been reported that CPP-ACP promoted the formation of fluorapatite deep in WSLs in the presence of fluoride but that CPP-ACP alone may not have this effect (12). In our study, the CPP-ACP did not have a significant effect on the color recovery of WSLs. This might be because the treatment consisted of one short application. Based on clinical trials recently reported, there is insufficient evidence to make a conclusion regarding the long- term effectiveness of casein derivatives such as CPP-ACP in preventing caries *in vivo* (16). Further studies investigating the long-term effects of CPP-ACP on the recovery of WSLs are needed.

Resin infiltration was originally developed to obstruct the diffusion pathways for acids in order to protect internal enamel, with a masking effect being an additional advantage (17). This study shows that it could be used to arrest incipient WSLs instead of removing the lesions, which sacrifices healthy enamel. Microabrasion, which has been commonly used for treating WSLs, can remove up to 250 mm of enamel (18). In contrast, in the clinical practice of resin infiltration; the 15% HCl gel used to prepare the surface and open the pores of WSLs removes only about 40 mm of the surface layer. In our study, no cavitation occurred after etching with the HCl gel, and the subsequent resin infiltration helped strengthen the WSL structures (19). Because resin infiltration can immediately restore the color of WSLs, it saves time for patients and clinicians. In addition, resin infiltration maintained color stability for at least 6 weeks after application in our study. These features indicate that resin infiltration was the best of the techniques tested for the treatment of WSLs, which is consistent with a previous study that showed that resin infiltration was an effective treatment for masking WSLs and resisting a new acid challenge (20). However, the long-term effect of resin infiltration on WSLs in clinical practice should be studied further. The BiominF and CPP-ACP + F treatment and remineralization times should be extended to determine how long the remineralization modalities take to restore the WSLs to a normal enamel appearance. In addition, a randomized controlled clinical trial comparing these different treatment modalities would provide valuable information for dental practitioners.

## Conclusions

Resin infiltration can improve the esthetic characteristics (color and fluorescence) of orthodontic WSLs; however, the long-term effect of resin infiltration on WSLs in clinical practice should be studied further.
